# Honokiol Enhances TRAIL-Mediated Apoptosis through STAMBPL1-Induced Survivin and c-FLIP Degradation

**DOI:** 10.3390/biom9120838

**Published:** 2019-12-06

**Authors:** Seon Min Woo, Seung Un Seo, Peter Kubatka, Kyoung-jin Min, Taeg Kyu Kwon

**Affiliations:** 1 Department of Immunology, Keimyung University, 1095 Dalgubeoldaero, Dalseo-Gu, Daegu 42601, Korea; woosm724@gmail.com (S.M.W.); sbr2010@hanmail.net (S.U.S.); kyoungjin.min@gmail.com (K.-j.M.); 2 Department of Medical Biology, Jessenius Faculty of Medicine, Comenius University in Bratislava, 03601 Martin, Slovakia; kubatkap@gmail.com; 3 Department of Experimental Carcinogenesis, Division of Oncology, Biomedical Center Martin, Jessenius Faculty of Medicine, Comenius University in Bratislava, 03601 Martin, Slovakia

**Keywords:** honokiol, TRAIL, c-FLIP, survivin, STAMBPL1

## Abstract

Honokiol is a natural biphenolic compound extracted from traditional Chinese medicine *Magnolia* species, which have been known to display various biological effects including anti-cancer, anti-proliferative, anti-angiogenic, and anti-metastatic activities in cancer cells. Here, we found that honokiol sensitizes cancer cells to tumor necrosis factor-related apoptosis-inducing ligand (TRAIL)-induced apoptosis through downregulation of anti-apoptotic proteins survivin and c-FLIP. Ectopic expression of survivin and c-FLIP markedly abolished honokiol and TRAIL-induced apoptosis. Mechanistically, honokiol induced protein degradation of c-FLIP and survivin through STAMBPL1, a deubiquitinase. STAMBPL1 interacted with survivin and c-FLIP, resulted in reduction of ubiquitination. Knockdown of STAMBPL1 reduced survivin and c-FLIP protein levels, while overexpression of STAMBPL1 inhibited honokinol-induced survivin and c-FLIP degradation. Our findings provided that honokiol could overcome TRAIL resistance through survivin and c-FLIP degradation induced by inhibition of STAMBPL1 expression.

## 1. Introduction

Even though tumor necrosis factor-related apoptosis-inducing ligand (TRAIL) is characterized by induction of death receptor (DR)-mediated apoptosis in cancer cells, most of cancer cells show resistance to TRAIL [1,2]. The typical factors of TRAIL resistance are downregulation of DRs (DR4 and DR5) and upregulation of decoy receptors (DcR1 and DcR2) [3,4]. In addition, overexpression of anti-apoptotic proteins, such as Bcl-2 family, IAP family, and c-FLIP, or downregulation of pro-apoptotic Bcl-2 family proteins decrease TRAIL-induced cancer cell death [5]. Therefore, to solve limitation of overcome to TRAIL tolerance in cancer therapy, many researchers have shown that combined treatment with chemotherapeutic agents can increase TRAIL sensitivity, and made an effort to identify TRAIL sensitizers [6,7].

Honokiol (a molecular formula of C_18_H_18_O_2_), one of bioactive biphenolic compound extracted from *Magnolia*, presents diversely biological functions such as anti-cancer, anti-angiogenesis, anti-inflammatory, and anti-oxidative properties in vitro and in vivo [8,9,10]. Previous studies investigated that honokiol increases mitochondrial dysfunction, resulting in induction of ROS-dependent apoptosis in cancer cells [11,12]. In addition, honokiol is regarded as sensitizer to increase anti-cancer effects of chemotherapeutic agents in various cancer cells. For example, honokiol sensitizes cancer cells to death receptor-mediated apoptosis through c-FLIP and Nur77 downregulation in lung and breast cancer cells, respectively [13,14]. Honokiol also overcomes resistance to chemotherapy and radiotherapy of many cancer cells through induction of apoptosis [15,16,17,18,19]. Moreover, induction of programmed necrotic cell death and paraptosis by honokiol affects in synergy to chemotherapy drugs [20,21]. Therefore, honokiol could be an attractive agent capable of overcoming chemotherapy resistance.

Ubiquitination is a process for post-translational modification of protein, and dysregulation of ubiquitination is closely related with cancer [22,23,24]. Ubiquitination is catalyzed by the enzymatic cascade (E1 activating, E2 conjugating, and E3 ligating enzymes) and ubiquitinated proteins are degraded by proteasome [25]. Contrastively, deubiquitination is the reverse process of ubiquitination that inhibits protein degradation through deubiquitinases (DUBs)-mediated depolymerization and removal of ubiquitin from target proteins [26]. Although many studies focused on modulation of ubiquitination-mediated protein stabilization through E3 ligases, recently, the roles of DUBs are emphasized [27]. In mammals, approximately 100 DUBs have been identified and classified into five classes based on the catalytic domain, including ubiquitin-specific proteases (USPs), ubiquitin carboxyl-terminal hydrolases (UCHs), ovarian tumor proteases (OTUs), Machado–Joseph disease proteases (MJDs), and Jab1/MPN/MOV34 metalloenzymes (JAMMs) [28,29]. STAM-binding protein-like 1 (STAMBPL1, also called AMSH-2 and AMSH-LP) belongs to family proteins of JAMM DUBs that cleaves Lys63 ubiquitin linkage [30,31]. Previous studies reported that although they did not investigate the DUB activity of STAMBPL1, STAMBPL1 interact with Smad2 and Smad7, followed by induction of TGF-β-mediated transcriptional activity [32]. In addition, STAMBPL1 stabilizes the human T-cell leukemia virus type 1 (HTLV-1) Tax oncoprotein [33]. Recently, we reported the depletion of STAMBPL1 increases apoptotic cell death through accumulation of intracellular ROS and lysosome-dependent XIAP degradation in prostate cancer cells [34], and we also reported that levels of STAMBPL1 is correlated with the expression of survivin in cepharanthine treated renal cancer cells [35]. However, the functions of STAMBPL1 and target proteins have not yet been understood.

Here, we investigated the effect of honokiol on the sensitization of cancer cells to anti-cancer drugs, and the underlying mechanism in cancer cells.

## 2. Materials and Methods

### 2.1. Cell Culture and Transfection

All cancer cells (Caki, A498, A549 and Hela) and TCMK-1 cells were obtained from American Type Culture Collection (Manassas, VA, USA). Human mesangial cells (MC) were purchased from Lonza (Basel, Switzerland). Cells were grown in appropriate medium supplemented with 10% FBS (Welgene, Gyeongsan, Korea), 1% penicillin-streptomycin, and 100 μg/mL gentamycin (Thermo Fisher Scientific, Waltham, MA, USA). For constructing stable cell lines, Caki cells were transfected using Lipofectamine^TM^ 2000 (Invitrogen, Carlsbad, CA, USA) with the pcDNA3.1(+)/Mcl-1, pcDNA3.1(+)/c-FLIP, pcDNA3.1(+)/survivin-flag or pcDNA3.1(+) vector plasmids. These plasmids were transduced for 24 h and cells were selected by 700 μg/mL G418 (Invitrogen, Carlsbad, CA, USA). For knockdown of genes by siRNA, Lipofectamine^®^ RNAiMAX Reagent (Invitrogen, Carlsbad, CA, USA) was used in Caki cells. Immunoblot analysis was performed to examine protein expression.

### 2.2. Reagents, Antibodies, siRNAs, and Plasmids

Sigma Chemical Co. provided honokiol, cycloheximide and MG132 (St. Louis, MO, USA), and R&D system supplied recombinant human recombinant TRAIL and z-VAD-fmk (Minneapolis, MN, USA). Enzo Life Sciences provided lactacystin (Ann Arbor, MI, USA). The primary antibodies were as follows: Cell Signaling Technology supplied anti-PARP, anti-cleaved caspase-3, anti-Bcl-xL, anti-DR5, anti-CHOP, and anti-UCHL1 (Beverly, MA, USA). Sigma Chemical Co. supplied anti-actin (St. Louis, MO, USA). Enzo Life Sciences provided anti-pro-caspase-3 and anti-c-FLIP (San Diego, CA, USA). BD Biosciences provided anti-Bim and anti-XIAP (San Jose, CA, USA). Abcam supplied anti-DR4 (Cambridge, MA, USA). R&D system supplied anti-survivin (Minneapolis, MN, USA). Santa Cruz Biotechnology provided anti-Mcl-1, anti-Bcl-2, anti-cIAP2, anti-ATF4, anti-Ub, anti-Cbl, anti-Itch, anti-USP14, anti-USP33, anti-OTUB1, anti-TRABID, and anti-STAMBPL1 (St. Louis, MO, USA). Bethyl Laboratories Inc provided anti-USP7 and anti-USP8 (Montgomery, TX, USA). Novus Biologicals supplied anti-USP53 (Centennial, CO, USA). Abnova provided anti-USP9X (Taipei City, Taiwan). The siRNAs were as follows: GFP (control) siRNA (Bioneer, Daejeon, Korea), DR5 siRNA (Invitrogen, Carlsbad, CA, USA), and STAMBPL1 siRNA (Santa Cruz Biotechnology, St. Louis, MO, USA). STAMBPL1 plasmid was a gift from Dr. H.C. Kang (Ajou University, Suwon, Korea).

### 2.3. FACS Analysis

For apoptosis analysis, cells were harvested and suspended in 100 μL of phosphate-buffered saline, and added to 200 μL of 95% ethanol [36]. And then, cells were incubated in 1.12% sodium citrate buffer containing RNase at 37 °C for 30 min, added to 50 μg/mL propidium iodide, and analyzed using BD Accuri™ C6 flow cytometer (BD Biosciences, San Jose, CA, USA).

### 2.4. Western Blotting

Cells were lysed in RIPA lysis buffer (20 mM HEPES and 0.5% Triton X-100, pH 7.6) and separated by 10% SDS-PAGE. Proteins were transferred to nitrocellulose membranes (GE Healthcare Life Science, Pittsburgh, PO, USA) and checked using an Immobilon Western Chemiluminescent HRP Substrate (EMD Millipore, Darmstadt, Germany) for analysis protein expression.

### 2.5. DNA Fragmentation and DEVDase Activity Assay for Detection of Apoptosis

Caki cells were treated with honokiol alone, TRAIL alone or honokiol plus TRAIL. To measure DNA fragmentation, we used cell death detection ELISA plus kit (Boehringer Mannheim, Indianapolis, IN, USA) according to the manufacturer’s recommendations. The reaction products were analyzed by spectrophotometry (BMG Labtech, Ortenberg, Germany) at 405 and 490 nm (reference wavelength). For DEVDase activity assay, cells were harvested and incubated with reaction buffer containing acetyl-Asp-Glu-Val-Asp p-nitroanilide (Ac-DEVD-pNA) substrate, as previously described [37].

### 2.6. Reverse Transcription Polymerase Chain Reaction (RT-PCR) and Quantitative PCR (qPCR)

Total RNA was isolated with TriZol reagent (Life Technologies, Gaithersburg, MD, USA), and prepared cDNA using M-MLV reverse transcriptase (Gibco-BRL, Gaithersburg, MD, USA). For PCR, we used Blend Taq DNA polymerase (Toyobo, Osaka, Japan) with primers targeting DR5, c-FLIP, survivin, and actin. The used primers were referred to previous studies [38,39]. For qPCR, SYBR Fast qPCR Mix (Takara Bio Inc., Shiga, Japan) was used, and reactions were performed on Thermal Cycler Dice^®^ Real Time System III (Takara Bio Inc., Shiga, Japan). We used STAMBPL1 and actin primers for qPCR: STAMBPL1 (sense) 5′-GGG ACC ATC GCA GTG ACA AT-3′ and (antisense) 5′-CCG ACA GAT GGA GCT TTG CT-3′, and actin (sense) 5′-CTA CAA TGA GCT GCG TGT G-3′ and (antisense) 5′-TGG GGT GTT GAA GGT CTC-3′. We calculated the threshold cycle number (Ct) of each gene using actin as the reference gene, and we reported the delta-delta Ct values of the genes.

### 2.7. Detection of DR5 Expression on Cell Surface

Detached cells by 0.2% EDTA were washed with PBS, and then suspended in 100 μM PBS including 10% FCS and 1% sodium azide, and added to the primary antibody (DR5-phycoerythrin, ab55863; Abcam, Cambridge, MA, USA) for 2 h at room temperature. Then, the cells washed with PBS including 10% FCS and 1% sodium azide, and were suspended in 400 μL of PBS for the detection of surface DR5 expression by flow cytometry.

### 2.8. Deubiquitination Assay

For deubiquitination assay, HA-Ubiquitin plasmid was transfected into Caki cells. After 24 h, the cells were pretreated with of MG132 for 6 h. Cells were harvested, washed with PBS containing 10 mM N-Ethylmaleimide (NEM), resuspended in 100 μL PBS/NEM containing 1% SDS, and boiled for 10 min at 95 °C. Lysates were added to RIPA lysis buffer involving 1 mM PMSF and 5 mM NEM, dissolved using l mL syringe for 3–4 times and centrifuged at 13,000× *g* for 10 min at 4 °C. The supernatants were incubated with the primary antibody of the target protein overnight and reacted by adding protein G agarose bead (Santa Cruz Biotechnology, St. Louis, MO, USA) for 2 h. After centrifuging, the supernatants were removed, washed with lysis buffer containing 1 mM PMSF and 5 mM NEM at 2 times and boiled using 2× sample buffer for 10 min. Ubiquitination assay were detected by Western blotting in denaturation condition with anti-Ub (BML-PW0150-0100, Enzo Life Sciences, San Diego, CA, USA).

### 2.9. Immunoprecipitation

To examine the interaction between STAMBPL1 and survivin/c-FLIP, immunoprecipitation was performed according to methods described in our previous study [40]. Briefly, cells were lysed in CHAPS lysis buffer and incubated with each primary antibody overnight. Lysates were reacted by adding protein G agarose beads for 2 h. After centrifuging, the supernatants were removed and boiled using the 2× sample buffer. Protein interaction was detected using Western blotting.

### 2.10. Statistical Analysis

The data were analyzed using a one-way ANOVA and post-hoc comparisons (Student-Newman-Keuls) using the SPSS software (SPSS Inc., Chicago, IL, USA).

## 3. Results

### 3.1. Honokiol Sensitizes Cancer Cells to TRAIL-Mediated Apoptosis, but Not Normal Cells

**Figure 1:**
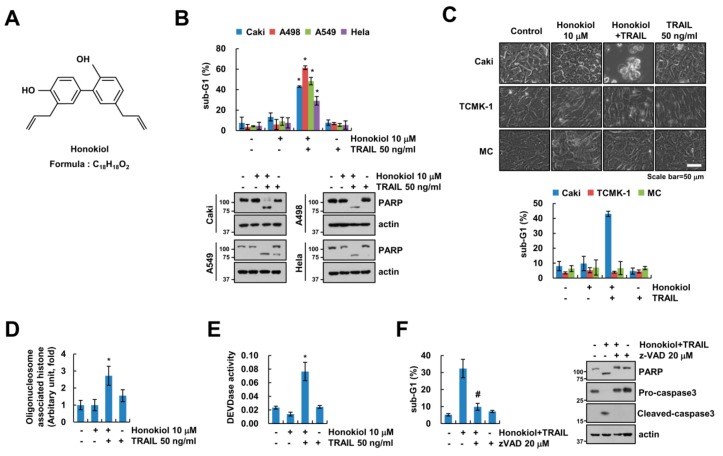
Honokiol enhances TRAIL-induced apoptosis. (**A**) Chemical structures of honokiol. (**B**) Indicated cancer cells were treated with 10 μM honokiol alone, 50 ng/mL TRAIL alone, or honokiol plus TRAIL for 24 h. (**C**) Caki and normal cells (TCMK-1 and MC) were treated with 10 μM honokiol, 50 ng/mL TRAIL, or honokiol plus TRAIL for 24 h. The cell morphology was examined using interference light microscopy. (**D**,**E**) Cytoplasmic histone-associated DNA fragments (**D**), and DEVDase (caspase-3) activity (**E**) were examined. (**F**) Caki cells were treated with 10 μM honokiol plus 50 ng/mL TRAIL in the presence or absence of 20 μM z-VAD for 24 h. The sub-G1 population and protein expression were detected by flow cytometry (**B**,**C**,**F**) and Western blotting (**B**,**F**), respectively. The values in graph (**B**–**F**) represent the mean ± SD of three independent experiments. * *p* < 0.01 compared to the control. # *p* < 0.01 compared to the honokiol and TRAIL. TRAIL: tumor necrosis factor-related apoptosis-inducing ligand.

In previous study, honokiol has anti-cancer effects in human lung cancer cells [14]. Therefore, we investigated whether sub-toxic concentrations of honokiol has synergy effects with anti-cancer drugs in renal carcinoma cells. Sub-toxic concentrations of honokiol alone and TRAIL alone did not induce cell death, but combined treatment dose-dependently increased cell death in renal carcinoma Caki cells (Supplementary Figure S1A). Moreover, honokiol sensitized cancer cells to TRAIL-mediated apoptotic cell death, but not normal cells (Figure 1B,C). In addition, we found that the nuclear chromatin condensation and DNA fragmentation were markedly increased in combined treatment with honokiol and TRAIL (Supplementary Figure S1B and Figure 1D). To examine the importance of caspase in apoptosis by combined treatment with honokiol and TRAIL, we checked caspase activities. Both honokiol plus TRAIL treatment activated caspase-3, -8, and -9 (Figure 1E and Supplementary Figure S1C,D). Furthermore, z-VAD-fmk (z-VAD), a pan-caspase inhibitor, completely blocked combined treatment-induced sub-G1 population, PARP cleavage and caspase-3 cleavage (Figure 1F). These data indicate that honokiol improves the efficacy of TRAIL-induced apoptosis in cancer cells.

### 3.2. Upregulation of DR5 by Honokiol Is Not Involved in Enhancement of TRAIL Sensitivity

**Figure 2:**
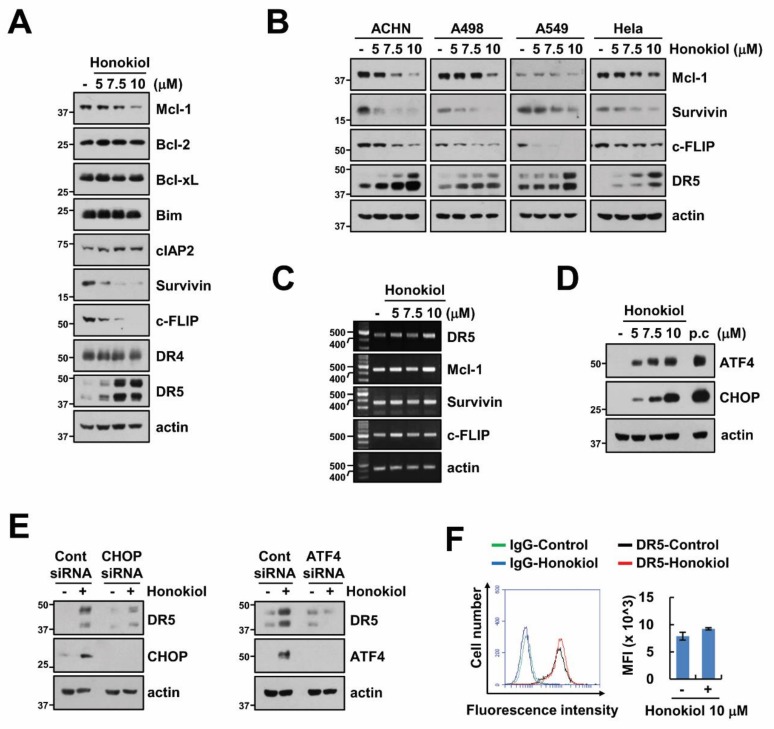
Upregulation of DR5 by honokiol is not involved in TRAIL-mediated apoptosis. (**A**–**D**) Caki cells (**A**,**C**,**D**) and indicated cancer cells (**B**) were treated with various concentrations of honokiol for 24 h (**A**–**C**) or 9 h (**D**). (positive control (p.c); 2 μM thapsigargin treatment for 9 h). (**E**) Caki cells were transfected with control (Cont), ATF4 or CHOP siRNA. Caki cells were treated with 10 μM honokiol for 24 h. (**F**) Analysis of DR5 expression on the cell surface. Caki cells were treated with 10 μM honokiol for 24 h and measured by flow cytometry analysis. The levels of mRNA were examined using RT-PCR (**C**). The protein expression was detected by Western blotting (**A**,**B**,**D**,**E**), respectively. The values in graph (**F**) represent the mean ± SD of three independent experiments.

Next, we screened alteration of apoptosis-related protein levels by honokiol, and found that honokiol increased DR5 expression and decreased Mcl-1, survivin, and c-FLIP expression in renal carcinoma (Caki, ACHN and A498), lung carcinoma (A549), and cervical cancer (Hela) cells (Figure 2A,B). However, honokiol only upregulated DR5 mRNA levels, and mRNA of others was not induced by honokiol treatment (Figure 2C). ER stress-related proteins, such as CHOP and ATF4, are involved in regulation of DR5 mRNA levels by acting as transcription factor [41,42], and honokiol induces endoplasmic reticulum (ER) stress through activation of CHOP [43,44]. Therefore, we investigated whether honokiol increased CHOP and ATF4 expression in our system. As shown in Figure 2D, ATF4 and CHOP expression were increased by honokiol treatment. To certify the involvement of ATF4 and CHOP on honokiol-induced upregulation of DR5, we used knockdown system using siRNA. As expected, knockdown of CHOP and ATF4 disturbed DR5 upregulation by honokiol (Figure 2E). Localization of DR5 on the cellular surface is a critical role in DRs-dependent extrinsic TRAIL-induced apoptosis, we examined expression of DR5 on the surface. However, honokiol did not alter surface DR5 expression (Figure 2F). Even though honokiol increased DR5 mRNA and protein expression, surface expression level of DR5 was not induced. Therefore, DR5 upregulation is not associated with honokiol-induced TRAIL sensitivity.

### 3.3. Downregulation of Survivin and c-FLIP Is Associated with Honokiol Plus TRAIL-Induced Apoptosis

**Figure 3:**
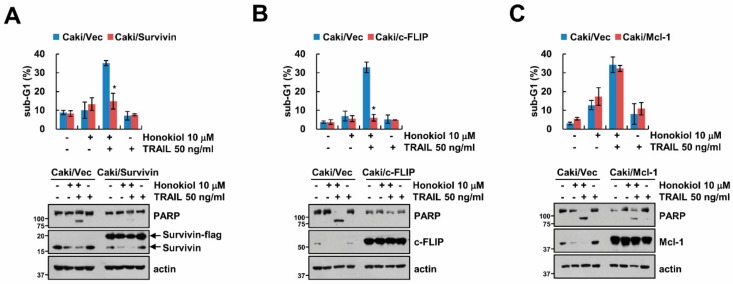
Overexpression of survivin and c-FLIP inhibits the induction of apoptosis by combined treatment with honokiol and TRAIL. (**A**–**C**) Vector cells, survivin- (**A**), c-FLIP- (**B**), and Mcl-1-overexpressing cells (**C**) were treated with 10 μM honokol in the presence or absence of 50 ng/mL TRAIL for 24 h. The sub-G1 population and protein expression were detected by flow cytometry and Western blotting, respectively (**A**–**C**). The values in graph (**A**–**C**) represent the mean ± SD of three independent samples. * *p* < 0.01 compared to honokiol plus TRAIL in Caki/Vec.

As shown in Figure 2A, since honokiol decreased Mcl-1, survivin and c-FLIP expression, we explored the role of these proteins on induction of TRAIL sensitivity by honokiol using survivin-, c-FLIP, and Mc1-1-overexpressed stable cells. Overexpression of survivin or c-FLIP markedly prevented sub-G1 population and PARP cleavage by honokiol plus TRAIL treatment (Figure 3A,B), while overexpression of Mcl-1 still induced apoptosis by combined treatment (Figure 3C). These results suggest that decrease of survivin and c-FLIP by honokiol contributes to induction of TRAIL sensitivity.

### 3.4. Honokiol Induces Survivin and c-FLIP Degradation through Activation of Ubiquitin-Proteasome System

**Figure 4:**
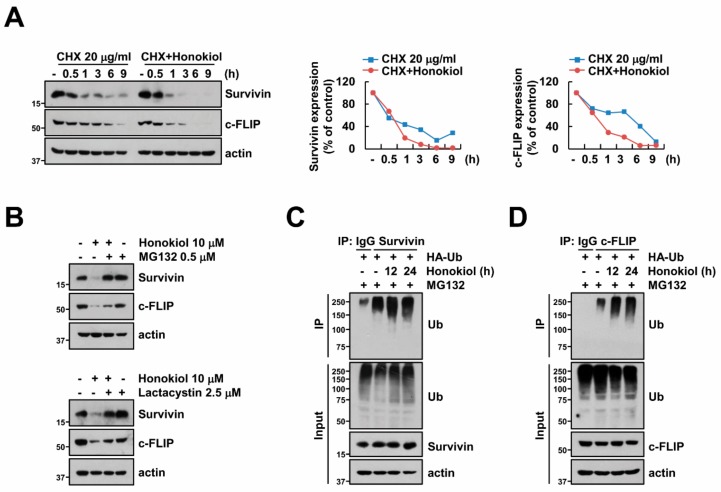
Honokiol degrades survivin and c-FLIP proteins by triggering ubiquitination. (**A**) Caki cells were treated with 10 μM honokiol in the presence or absence of 20 μM CHX for the indicated time kinetics. The band intensity was quantified using Image J. (**B**) Caki cells were treated with 10 μM honokiol in the presence or absence of 0.5 μM MG132 or 2.5 μM lactacystin for 24 h. (**C**,**D**) To analyze the ubiquitination of endogenous survivin (**C**) and c-FLIP (**D**), Caki cells were transfected with HA-ubiquitin (HA-Ub) plasmid and treated with 0.5 μM MG132 plus 10 μM honokiol for the indicated time kinetics. Protein ubiquitination was analyzed by Western blotting using anti-survivin (**C**) and c-FLIP (**D**) antibodies for immunoprecipitation (IP). Protein expression was detected by Western blotting (**A**–**D**).

Next, because honokiol induced downregulation of survivin and c-FLIP protein expression (Figure 2A,B), we examined the decrease of these proteins through post-translational regulation. First, we checked survivin and c-FLIP protein stability in the presence of cycloheximide (CHX), an inhibitor of protein biosynthesis. Honokiol more quickly degraded suvivin and c-FLIP expression compared to CHX (Figure 4A). Previous studies presented that ubiquitin-proteasome system (UPS) is critical role to protein stabilization and degradation [22,23]. Therefore, we examined whether honokiol-induced down-regulation of survivin and c-FLIP is dependent on proteasome activity using proteasome inhibitors (MG132 and lactacystin). Proteasome inhibitors reversed survivin and c-FLIP downregulation by honokiol (Figure 4B). These data indicate that honokiol decreases survivin and c-FLIP expression via degradation of proteins.

Previous studies presented that many proteins including survivin and c-FLIP are degraded through activation of ubiquitination [45,46,47]. To confirm the effect of ubiquitination on degradation of survivin and c-FLIP expression, we analyzed ubiquitination of survivin and c-FLIP by honokiol. When immunoprecipitation conducted with survivin or c-FLIP antibodies under denaturing conditions, honokiol led to increase ubiquitination of endogenous survivin and c-FLIP (Figure 4C,D). Therefore, these data suggest that honokiol could degrade survivin and c-FLIP protein expression via ubiquitination-proteasome system.

### 3.5. STAMBPL1 Can Regulate Survivin and c-FLIP Stability

**Figure 5:**
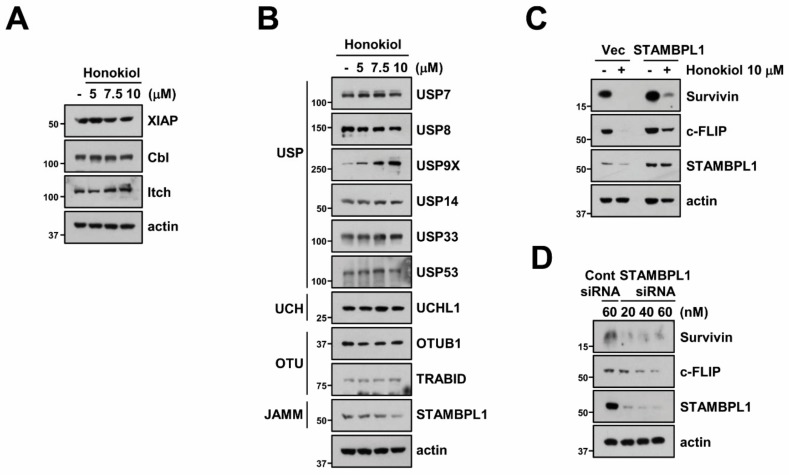
STAMBPL1 is associated with honokiol-mediated downregulation of c-FLIP and survivin. (**A**,**B**) Caki cells were treated with various concentrations of honokiol for 24 h. (**C**) Caki cells were transfected with pcDNA3.1 (+) vector or STAMBPL1 plasmid and then treated with 10 μM honokiol for 24 h. (**D**) Caki cells were transfected with control (Cont) or STAMBPL1 siRNA for 24 h, and then cells were further incubated for 24 h. Protein expression were detected by Western blotting (**A**–**D**).

Activation of E3 ligases plays a major role in proteasome-mediated protein degradation by attaching of ubiquitin from E2 conjugating enzymes to substrate [48]. We investigated whether honokiol can regulate E3 ligase of survivin (XIAP) and c-FLIP (Cbl and Itch). However, three E3 ligases were not modified by honokiol (Figure 5A). Therefore, we tested alteration of various DUBs expression and discovered that honokiol only decreased STAMBPL1 which is classified as JAMM family (Figure 5B). To further verify the involvement of STAMBPL1 in honokiol-induced survivin and c-FLIP degradation, we overexpressed STAMBPL1 in Caki cells. Ectopic expression of STAMBPL1 reversed survivin and c-FLIP degradation by honokiol treatment (Figure 5C). Moreover, silencing of STAMBPL1 by siRNA induced survivin and c-FLIP downregulation (Figure 5D). These findings suggest that STMABPL1 is a novel DUBs that can regulate survivin and c-FLIP stability.

### 3.6. STAMBPL1 Interacts and Induces Deubiquitination of Survivin and c-FLIP

**Figure 6:**
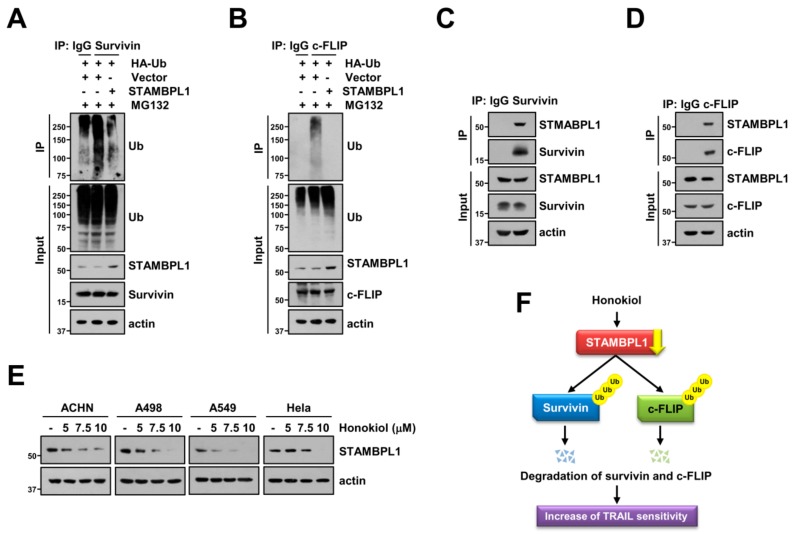
STAMBPL1 interacts with survivin and c-FLIP proteins, and modulates their stability. (**A**,**B**) To analyze the ubiquitination of endogenous survivin (**A**) and c-FLIP (**B**), Caki cells were transfected with pcDNA3.1 (+) vector or STAMBPL1 plasmid in the presence of HA-ubiquitin (HA-Ub) and plasmid, and then treated with 0.5 μM MG132 for 6 h. Protein ubiquitination was analyzed by Western blotting using anti-survivin (**A**) and c-FLIP (**B**) antibodies for immunoprecipitation (IP). (**C**,**D**) Endogenous STAMBPL1 and survivin or c-FLIP were immunoprecipitated from Caki cells using anti-survivin (**C**) or anti-c-FLIP (**D**) antibodies, respectively. (**E**) Indicated cancer cells were treated with various concentrations of honokiol for 24 h. Protein interaction (**C**,**D**) and expression (**A**–**E**) were analyzed by Western blotting. (**F**) Scheme indicating the mechanism to increase TRAIL sensitivity by honokiol.

Next, we explored deubiquitination of survivin and c-FLIP by STAMBPL1. Overexpression of STAMBPL1 reduced ubiquitination of endogenous survivin and c-FLIP (Figure 6A,B). DUBs can directly interact with target proteins, followed by regulation of cellular processes [29,49]. Therefore, we speculated that STAMBPL1 can directly bind to survivin and c-FLIP proteins. As shown in Figure 6C, we detected the interaction between STAMBPL1 and survivin. In addition, STAMBPL1 also bound endogenous c-FLIP in Caki cells (Figure 6D). To popularize phenomenon by honokiol treatment, we examined the alteration of STAMBPL1 by honokiol in other cancer cells. Honokiol induced STAMBPL1 downregulation in tested cancer cells (ACHN, A498, A549, and Hela cells) (Figure 6E). Thus, these data reveal that STAMBPL1 directly interacts with survivin and c-FLIP, resulting in stabilization of proteins.

## 4. Discussion

In this study, we demonstrated that honokiol increased TRAIL sensitivity of cancer cells through survivin and c-FLIP downregulation. For the first time, we identified STAMBPL1 as a novel deubiquitinase which interacts with and inhibits ubiquitination of survivin and c-FLIP. Honokiol decreased STAMBPL1 expression, resulting in degradation of survivin and c-FLIP protein. Moreover, overexpression of STAMBPL1 reversed honokiol-induced survivin and c-FLIP degradation. Therefore, honokiol-induced STAMBPL1 downregulation is a critical role in sensitization cancer cells to TRAIL-mediated apoptosis via degradation of survivin and c-FLIP (Figure 6F).

Renal cell carcinoma (RCC) is the third most common cancer in United States, and is classified by histological subtypes, such as clear cell RCC (ccRCC) (~80%), papillary RCC (pRCC) (~15%), and chromophobe RCC (chRCC) (~5%) [50]. ccRCC (Caki and A498 cells) is most often mutated von Hippel–Lindau and c-Met genes, whereas pRCC (ACNH cells) is presented mutation of PBRM1 gene [51,52,53]. Therefore, RCC is characterized by metastatic and uncontrolled cell proliferation, followed by resistance to usual chemotherapies [54]. Even though most common RCC is indicated drug resistance by gene mutations, honokiol increased TRAIL sensitivity in two subtypes of RCC (Figure 1B and unpublished data). Thus, our results suggest that honokiol overcomes drug resistance regardless of gene mutation in RCC.

Recently, Zhu et al. reported that honokiol induces ER stress-dependent apoptosis via CHOP upregulation in human lung cancer cells, and knockdown of CHOP blocks honokiol-induced caspase 9 activities [44]. In our study, honokiol also increased CHOP and ATF4 expression (Figure 2C), but downregulation of CHOP and ATF4 using siRNA did not inhibit honokiol plus TRAIL-induced apoptosis (Supplementary Figure S2A,B). Zhu et al. used high concentrations of honokiol (60 μM), thus honokiol alone increased apoptosis [44]. However, we used low concentrations of honokiol (10 μM), which did not induce apoptosis (Figure 1B). ER stress-induced apoptosis is dependent on duration and intensity of ER stress [55]. Although low concentrations of honokiol induce ER stress, intensity and duration might not be enough to induce apoptosis. Thus, we rule out the relevance of ER stress in anti-cancer effects by honokiol.

In previous studies, honokiol downregulates survivin and c-FLIP expression [14,56], and enforced expression of these proteins diminishes apoptosis and sensitivity cancer cells to anti-cancer drug by honokiol. However, these reports did not investigate the underlying molecular mechanisms of downregulation of survivin and c-FLIP. We also found that honokiol decreased survivin and c-FLIP expression at post-translational level (Figure 4A). To confirm the involvement of UPS in survivin and c-FLIP downregulation by honokiol, we used proteasome inhibitors (MG132 and lactacystin) and performed ubiquitination assay. Honokiol induced ubiquitination of survivin and c-FLIP, and proteasome inhibitors blocked honokiol-mediated survivin and c-FLIP downregulation (Figure 4B–D). Therefore, these data indicated that honokiol degrades survivin and c-FLIP proteins via ubiquitin-proteasome pathways.

Degradation of proteins could be regulated by activation of E3 ligases. XIAP acts as E3 ligases of survivin [57], and Cbl and Itch act as E3 ligases of c-FLIP [58,59]. However, honokiol did not alter these E3 ligases expression level (Figure 5A). Therefore, we focused alteration of DUBs expression by honokiol. In a previous study, Jeong et al. reported that c-FLIP is stabilized by USP8, followed by suppression of death receptor-mediated apoptosis [60]. However, honokiol had no effect on expression of USP8 in renal carcinoma Caki cells (Figure 5B). In case of survivin, USP9X and STAMBPL1 modulate survivin expression by long noncoding RNA (lncRNA) LNC473 and cepharanthine, respectively [35,61]. LNC473 directly bind survivin and USP9X, and USP9X inhibits ubiquitination of survivin [61]. However, although cepharanthine reduces STAMBPL1 expression and overexpression of STAMBPL1 inhibits cepharanthine-mediated downregulation of survivin, interaction between STAMBPL1 and survivin was not investigated. Here, we found that STAMPL1 directly binds survivin, and modulates ubiquitination of survivin (Figure 6A,C). Honokiol dramatically inhibits STABMPL1 expression, but not USP9X (Figure 5B and Figure 6E). This means that there is probably a variety of DUBs rather than a single DUB that controls ubiquitination of target proteins, and that modulation of DUBs expression depending on stimuli could be control the stability of the target protein. In our study, the honokiol inhibited STAMBPL1 expression (Figure 5B).

Collectively, we showed that honokiol sensitizes cancer cells to TRAIL-induced apoptosis through STAMBPL1-mediated survivin and c-FLIP downregulation. Moreover, our findings provide the role of STABMPL1 in ubiquitin-dependent survivin and c-FLIP degradation.

## 5. Conclusions

Our study provides the evidence that honokiol sensitizes cancer cells to TRAIL-induced apoptosis through downregulation anti-apoptotic proteins, survivin and c-FLIP. Downregulation of c-FLIP and survivin protein is caused by honokiol-induced inhibition of STAMBPL1 (deubiquitinase) expression. Therefore, honokiol may represent an attractive sensitizer in TRAIL-resistant cancer cells.

## Supplementary Materials

The following are available online at https://www.mdpi.com/2218-273X/9/12/838/s1, Figure S1: The effect of honokiol on TRAIL-mediated caspase-dependent apoptosis, Figure S2: Activation of ER stress is not involved in combined treatment with honokiol plus TRAIL-induced apoptosis.
