# Supplementary figures and images for: Honokiol Enhances TRAIL-Mediated Apoptosis through STAMBPL1-Induced Survivin and c-FLIP Degradation
Source: Biomolecules. 2019 Dec 6;9(12):838. doi: 10.3390/biom9120838 (PMC6995549; doi:10.3390/biom9120838)

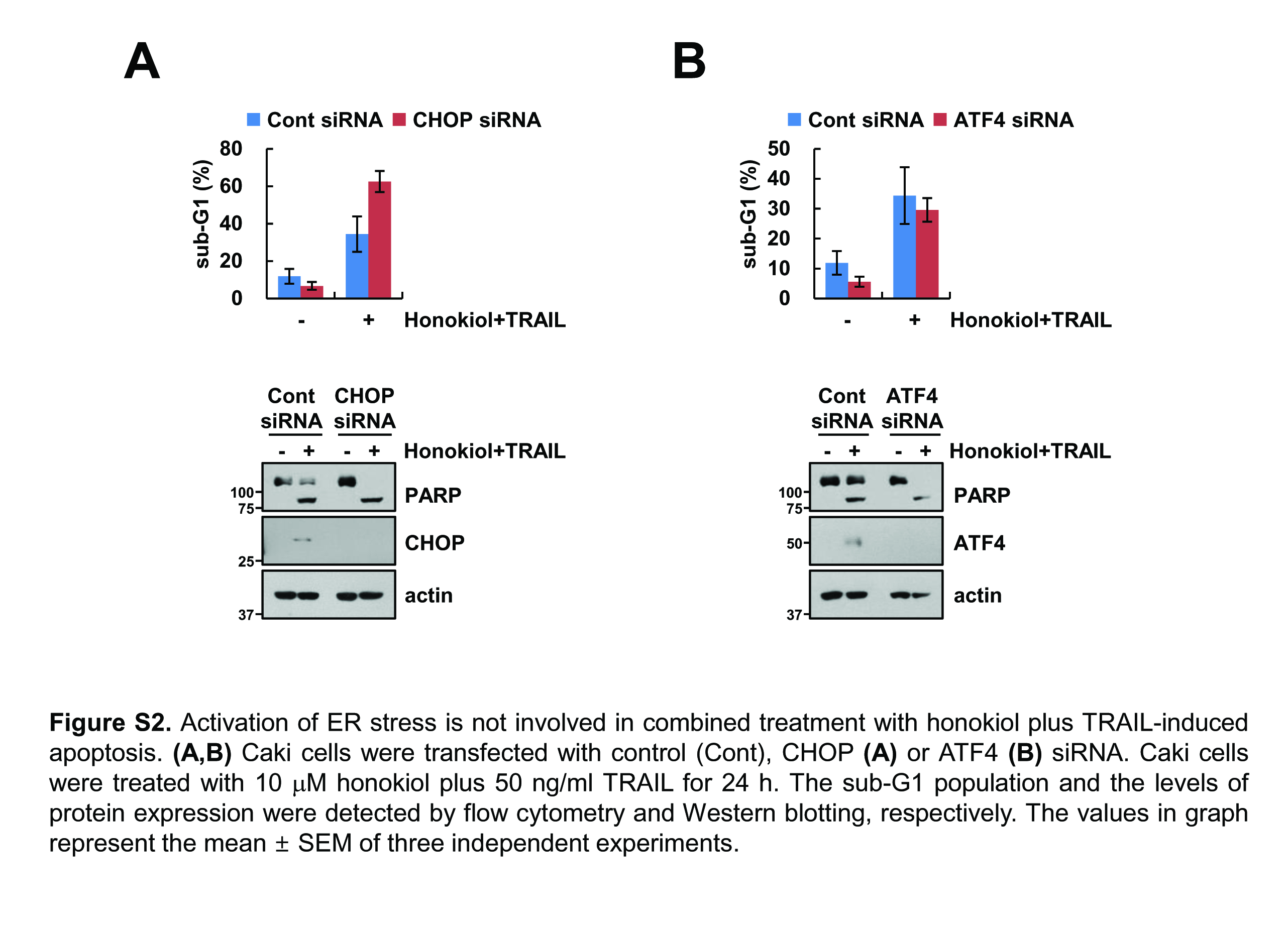

Supplement: Supplementary file 1 [file biomolecules-09-00838-s001.zip › Figure S2.tif]

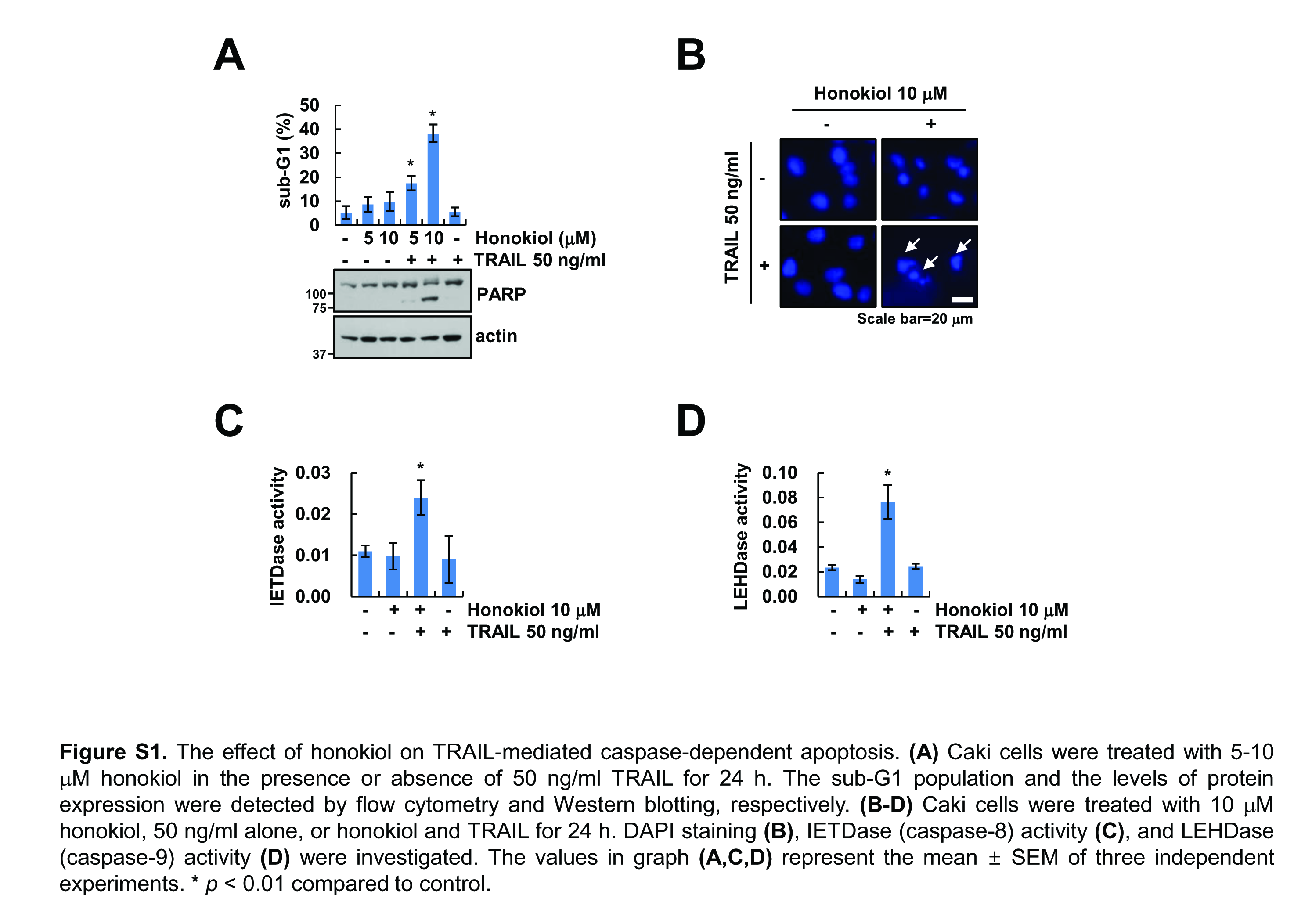

Supplement: Supplementary file 1 [file biomolecules-09-00838-s001.zip › Figure S1.tif]
